# Structural, Electronic, and Magnetic Properties of the van der Waals ScSi_2_N_4_/VSi_2_N_4_ Heterostructure: A First-Principles Study

**DOI:** 10.1021/acsomega.5c02195

**Published:** 2025-05-20

**Authors:** Brandon Pedroza-Rojas, Ariadna Sanchez-Castillo, Rodrigo Ponce-Pérez

**Affiliations:** † Instituto de Ciencias Básicas e Ingeniería, Área Académica de Ciencias de la Tierra y Materiales, Universidad Autónoma del Estado de Hidalgo, Carretera Pachuca-Tulancingo Km. 4.5, Mineral de la Reforma, Pachuca, Hidalgo CP 42184, México; ‡ Escuela Superior de Apan, Universidad Autónoma del Estado de Hidalgo, Carretera Apan-Calpulalpan Km. 8, Col Chimalpa, Apan, Hidalgo CP 43920, México; § Centro de Nanociencias y Nanotecnología, Universidad Nacional Autónoma de México, Ensenada CP 22800, BC, México

## Abstract

The van der Waals (vdW) heterostructures provide advantages compared to conventional interfaces, such as a well-defined interface region, being easy to construct, and reducing the stress between their components. This work uses spin-polarized first-principles calculations to investigate the van der Waals heterostructure based on MSi_2_N_4_ with M = Sc or V. Isolated monolayers exhibit ferromagnetic characteristics with half-metal or semiconductor behavior, respectively. Phonon calculations also show evidence of their dynamic stability. The heterostructure is investigated by considering three different stackings: Top, T4, and H3. Our calculations demonstrate that T4 stacking is the most stable configuration. Also, the noncovalent interactions index shows that only vdW forces participate. The magnetic coupling between monolayers is investigated. Our results show that ferromagnetic and antiferromagnetic coupling could appear in the experiment. Regarding electronic properties, the vdW heterostructure is metallic when the Sc layer is under compressive strain. However, the heterostructure becomes a half-metal if VSi_2_N_4_ is under tensile strain. Finally, the magnetic anisotropy energy is investigated. According to the results, the heterostructure has an in-plane magnetization axis in-plane. Our findings demonstrate that the magnetic intrinsic nature of the heterostructure is suitable for its implementation in spintronic devices such as spin valves.

## Graphical abstract

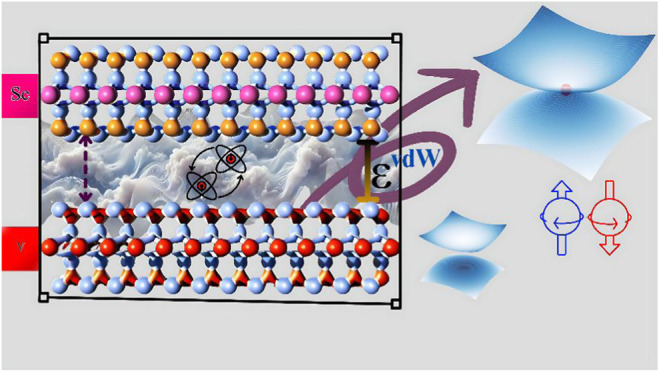

## 1 Introduction

Silicon-based spintronic materials have been displaced due to the challenges that typically arise when attempting to enhance the performance of their nanodevices.^1^ In recent years, two-dimensional (2D) materials with intrinsic magnetic properties have been explored for use in spintronic applications to reduce the size of the devices. Recently, a new family of 2D materials called MA_2_Z_4_ has been discovered. With M being an early transition metal, A becoming Si or Ge, and Z = N, P, As, or S superficial atoms. These kinds of materials were discovered when Hong et al. tried to dope Mo_2_N with Si during chemical vapor deposition (CVD) growth. Surprisingly, Si is not introduced as an imperfection generating this new family.^2,3^ The MoSi_2_N_4_ and WSi_2_N_4_ are unique compounds experimentally synthesized, which have attracted attention because of their magnetic properties. Also, the band offsets can be modulated with atomic adsorption, doping, vacancy,^4^ biaxial strain,^5–7^ hybrid heterostructures,^8–12^ and Janus frameworks.^13,14^ Besides, these compounds have been proposed to be employed in catalysis,^15^ electronics and optoelectronics,^16^ energy storage, and green energies,^17^ among others.

When the M element is Sc or V, the compounds are ferromagnetic (FM). The VSi_2_N_4_ compound, which has been predicted to be stable by density functional theory (DFT) calculations, has shown tunable valleys, gapless semiconducting,^18^ and spin splitting under electric field subjection in a bilayer stacking,^16^ a relevant fact to degenerate and control spin states.^19–21^ Also, this compound has been proposed as an anode material in Li-ion batteries.^22^ However, the most promising aspect of this monolayer (ML) is the recent substitutional doping enhancement for spin filtering^23^ and giant mobility in the spin-up channel.^24^

On the other hand, ScSi_2_N_4_ joined below van der Waals (vdW) forces to CuInP_2_S_6_—a ferroelectric 2D system—has shown electron transfer owing to compressive and tensile strain between S and N atoms, modulating the electronic behavior from half-metal to metal.^25^ In the case of Sc-based materials, magnetism comes from the nitrogen atoms bound to Sc, while in the V-based compounds, the origin of FM comes from the V atoms.^16^ Their magnetic properties become suitable for their implementation in spintronic, valleytronic, and spinorbitronic applications.^26^

van der Waals (vdW) heterostructure offers advantages compared to conventional interfaces, such as an atomically flat interface, strong proximity effects, and the possibility to twist the angle between layers.^27–29^ Besides, suppose that the vdW heterostructure is formed by intrinsic magnetic materials. In that case, it is possible to modulate their magnetic properties to become suitable for their implementation in spin filter tunneling,^30^ magnetic tunnel junctions,^31^ and spin–orbit-torque memory devices.^32,33^ Motivated by these possible applications, we investigated the structural, electronic, and magnetic properties of the vdW heterostructure formed by the VSi_2_N_4_ and ScSi_2_N_4_ compounds. Our findings demonstrate that the heterostructure is feasible with T4 stacking. By 3% tensile strain in the V-based layer, the heterostructure can acquire half-metal characteristics, while if the Sc-based layer is under −3% compressive strain, the heterostructure becomes metallic. The manuscript is organized as follows: Section 2 is for the computational details; Section 3 shows the results; and finally, in Section 4, the conclusions are made.

## 2 Computational Details

The vdW heterostructure formed by ScSi_2_N_4_ and VSi_2_N_4_ compounds was investigated by using spin-polarized first-principles calculations within the density functional theory (DFT) framework, as implemented in the Vienna Ab Initio Simulation Package (VASP)^34–36^ code. We employed the Projector Augmented Wave (PAW) method^37,38^ with 500 eV as the energy cutoff to describe the electron–ion interactions. The exchange–correlation energy is modeled by the Generalized Gradient Approximation (GGA) using the Perdew–Burke–Ernzerhof (PBE) parametrization.^39^ Besides, the vdW forces were considered by employing the DFT-D3 method of Grimme.^40,41^ Since V has d-electrons, the Hubbard correction (GGA + *U*) is considered with *U*_eff_ = 3 eV,^23,24,42^ following the simplified (rotationally invariant) approach introduced by Dudarev.^43^ The supercell method is employed to construct the vdW heterostructure. A 2 × 2 periodicity forms each supercell to avoid periodic ghost effects. Besides, the vacuum space is tested, finding that a vacuum space larger than 10 Å is enough to avoid interactions between periodic slabs; see Figure S1 from the Supporting Information (SI). Therefore, we choose a vacuum space larger than 20 Å in the heterostructure since we are modulating the interlayer distance up to 10 Å. In geometry optimization, all force components and energy differences must be less than 0.01 eV/Å and 1 × 10^–4^ eV, respectively. The Brillouin zone was sampled with a k-point grid of 8 × 8 × 1 according to the Monkhorst–Pack scheme.^44^ The noncovalent interactions (NCI) were investigated using the critic2 software.^45,46^ The stability of the structures was investigated by phonon calculations, employing the finite difference method^47^ and Phonopy code.^48^ We also investigated magnetic anisotropy energy (MAE) by performing noncollinear calculations. To do this, we take into consideration the spin–orbit coupling (SOC) effects.

We investigated the thermal stability of the heterostructure using ab initio molecular dynamics (AIMD) simulations carried out in the NVT ensemble. The Nose–Hover thermostat at 500 K was used with a step value of 1 fs 1000 times.

## 3 Results and Discussion

### 3.1 ScSi_2_N_4_ and VSi_2_N_4_ Layers

The structural, electronic, and magnetic properties of the VSi_2_N_4_ and ScSi_2_N_4_ compounds were initially investigated. VSi_2_N_4_ has a calculated cell parameter *a* = 2.87 Å, in agreement with previous reports,^7,10,15,16,22,49–52^ with an N–V bond distance of 2.03 Å. On the other hand, ScSi_2_N_4_ provides calculated cell parameters *a* = 2.96 Å with a Sc–N bond distance of 2.17 Å in concordance with previous reports.^13,16,19,23^ Figure 1a,1b shows the atomistic representations of both compounds. In both cases, magnetism is observed: the V-based system has a total magnetization of 0.88 μ_B_/unit cell, where V has a magnetic moment of 1.07 μ_B_, and neighboring N atoms acquire induced magnetization of −0.10 μ_B_; the Sc-based layer has a total magnetization of 0.90 μ_B_/unit cell, in these cases, each N bond to Sc atom provide a magnetization of 0.41 μ_B_. Also, Sc provides a small magnetization of 0.06 μ_B_. Besides, the electronic properties are investigated by computing their density of states (DOS) and band structure along the Γ–M–K−Γ path. The results are in Figure 1c,d. In all cases, the energy reference is the Fermi level. According to the results, ScSi_2_N_4_ is half-metal, whereas spin-up has a band gap of 2.34 eV, and spin-down behaves as a metal with the N-p orbitals as the main contribution to the DOS around the Fermi level, followed by the Sc-d orbitals. On the other hand, VSi_2_N_4_ behaves as a semiconductor, with band gaps of 0.54 and 1.95 eV for spin-up and down, respectively, with a narrow contribution of the V-d orbitals around the Fermi level. Zhan et al.^53^ reported semiconductor behavior around 0.50 eV for VSi_2_N_4_ by employing the HSE06 hybrid functional, which agrees with our findings. It is worth mentioning that Hong et al.^2^ reported the band gap of the MoSi_2_N_4_ compound by experimental and theoretical methods, finding an experimental energy gap of 1.94 eV and a theoretical value of 1.74 (2.30) eV by employing a GGA (HSE06) functional. Thus, we think that the DFT + *U* approach is enough to provide a good description of the electronic properties of the compounds.

**Figure 1:**
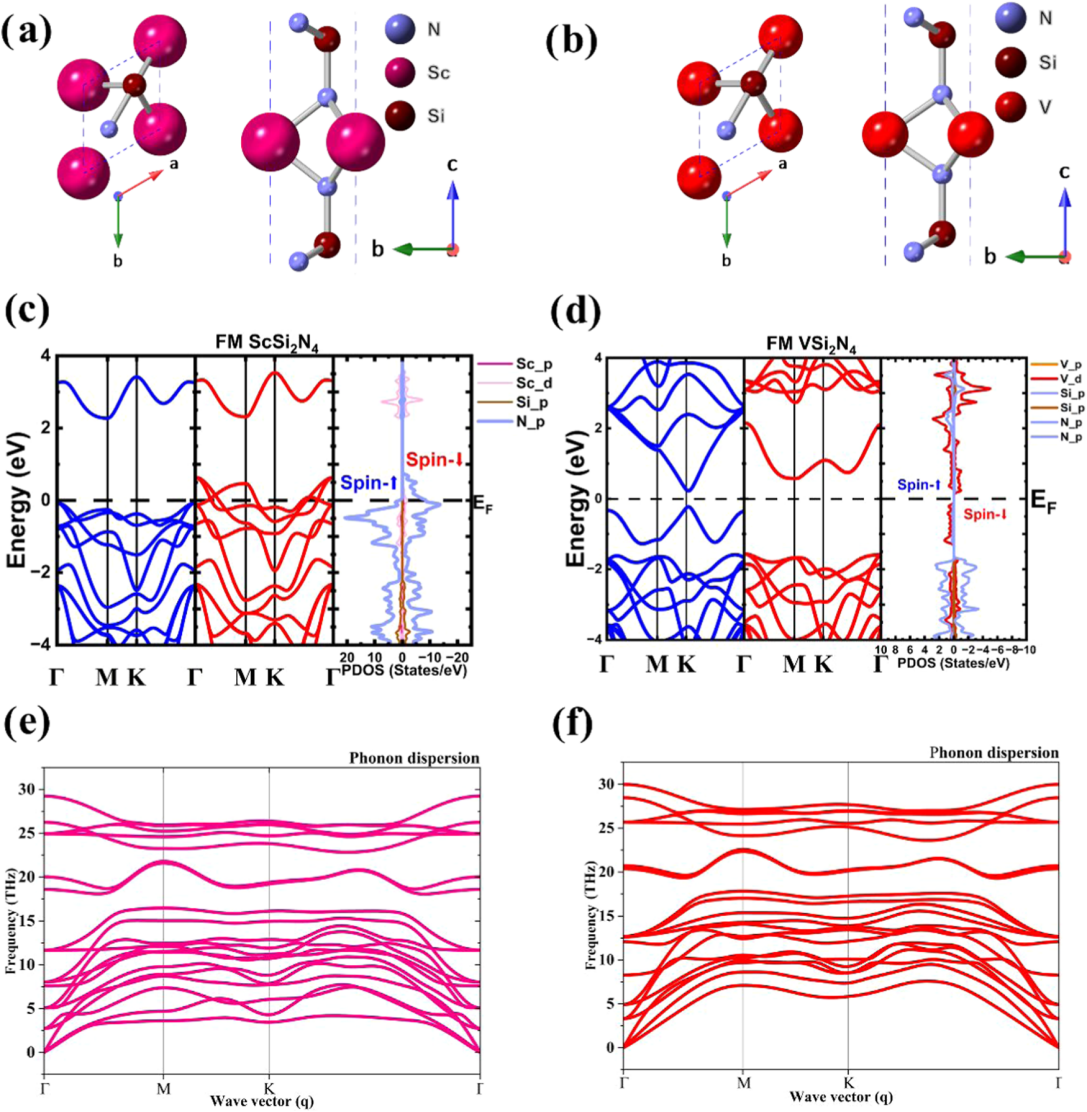
Top and side views of the (a) VSi_2_N_4_ and (b) ScSi_2_N_4_ compounds. Their corresponding band structures and DOS are in (c) and (d). Their phonon band structures are depicted in parts (e) and (f).

Besides, it is known that spin–orbit coupling (SOC) can be critical for structures with heavy atomic weights. In Figure S2 from the SI, we include the band structure of both compounds, considering the SOC effects. Our results show nonsignificant changes in the bands. Therefore, we can conclude that the SOC does not need to describe these structures. Finally, the dynamic stability of the monolayers (ML) is investigated, and the results reveal that all phonon branches are positive, demonstrating their stability, as shown in Figure 1e,1f.

### 3.2 vdW Heterostructure

The V- and Sc-based compounds have a lattice mismatch of ∼3%. Therefore, the formation of a vdW heterostructure is feasible. We considered three different stackings to construct the heterostructure, as shown in Figure 2, in a 2 × 2 periodicity. Also, we only consider the case when the heterostructure adopts the VSi_2_N_4_ cell parameter (ScSi_2_N_4_ is under −3% compressive strain) since the structural and magnetic properties are similar to the case when the V-based layer is under 3% tensile strain to adjust to the Sc-based layer cell parameter. The reference is the VSi_2_N_4_ ML (inner layer) in all cases. We were inspired by the interplay between the carbon layer in the graphite structure^54^ for H3 stacking, which corresponds to placing the Sc atoms on top of the N atoms of the V-based compound; T4 stacking occurs when the Si atoms of the upper layer are on top of the N atoms of the inner layer, same as grain boundaries instabilities are formed in molybdenum disulfide.^55^ The Top stacking occurs when the N atoms of the upper and lower layers are aligned, which is an AA alignment.

**Figure 2:**
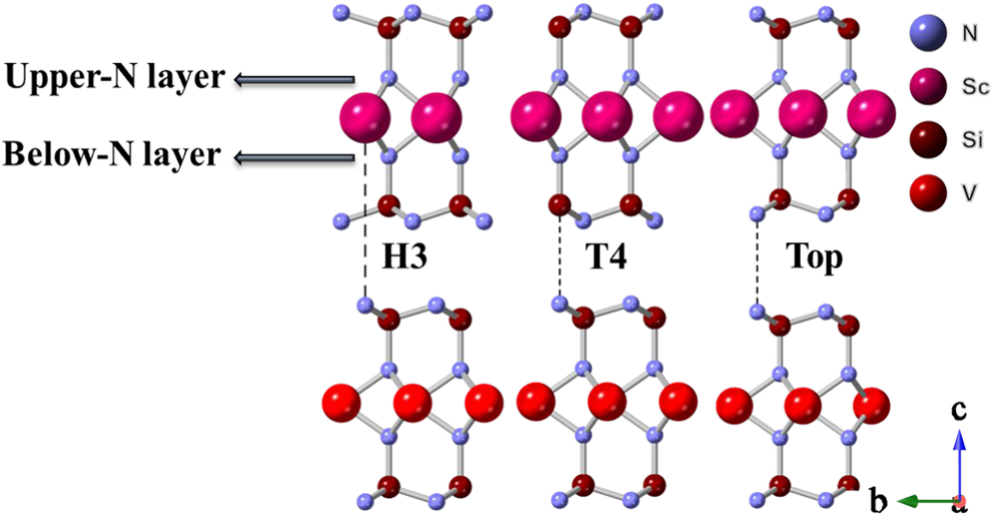
Side views of the three stackings employed in this work to construct the vdW heterostructure.

Once the different stackings were defined, the interlayer distance between them was varied until the total energies reached a minimum. The results are displayed in Figure 3. In all cases, the energy reference is the energy of both layers without interaction.

**Figure 3:**
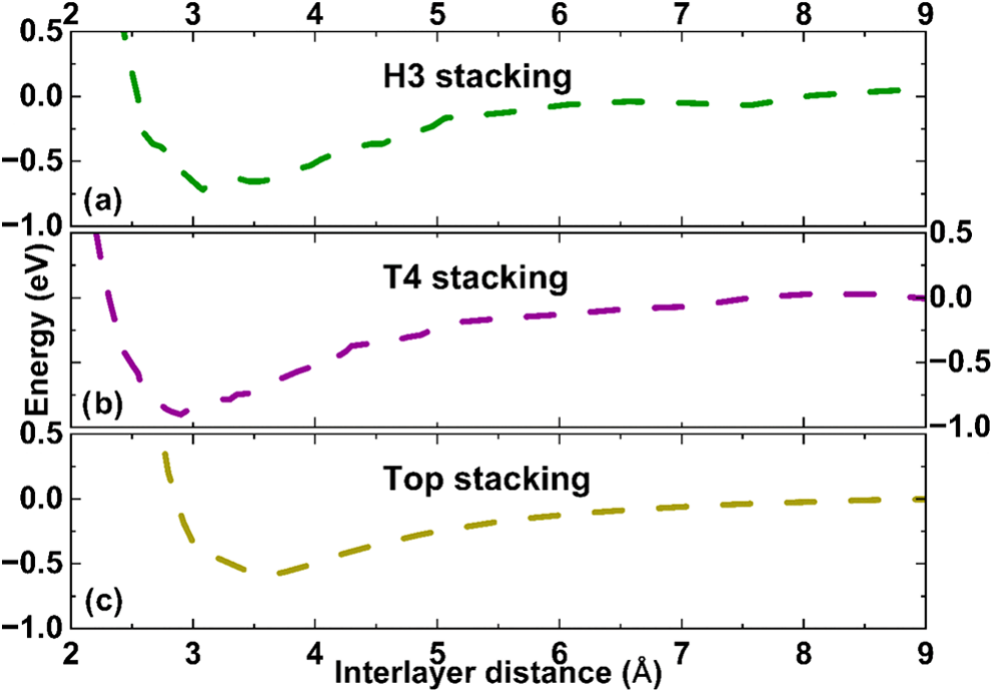
Interaction potential for (a) H3, (b) T4, and (c) Top stackings considered in this work.

The interlayer distances that reach the minimum energy are 2.92, 2.90, and 3.56 Å for the H3, T4, and Top arrangements, respectively. According to the results, the T4 stacking is the most stable configuration with a gain of energy of 771 meV compared with isolated monolayers. The H3 and Top configurations are less stable at 183 and 290 meV, respectively.

The noncovalent interaction (NCI) index is investigated to get a deep insight into the interlayer interaction in the three stackings.^45^ Figure 4a shows the reduced density gradient (*s*) as a function of the sign of the second eigenvalue of the Hessian of the density (λ_2_) multiplied by the electron density (ρ). In the graph, we focus on the region of low s and low ρ since the noncovalent interactions are located in this zone. The sign of λ_2_ describes the kind of interactions present in our systems; negative values (λ_2_ < 0) denote attractive interactions, while positive values (λ_2_ > 0) suggest repulsive interactions. Also, values close to zero (λ_2_ ∼ 0) denote weak interactions such as van der Waals forces. In all cases, singularities associated with bonds between layers are not observed. Only vdW interactions are noticed. Besides, Figure 4b–d shows the atomistic representation of the heterostructures, including the *s* isosurface (*s* = 0.5 au) colored in the RGB scheme. The red color is for repulsive interactions, blue is for attractive interactions, and green is for vdW interactions. In all cases, nonbond formation is noted at the interface. About Top stacking, the NCI shows the lowest interaction between layers, denoted by the green isosurface. In the H3 case, the larger isosurface is noticeable, which is mainly governed by vdW forces and some regions of repulsive interactions due to the green and yellow color of the isosurface. However, in the T4 stacking, although it shows a smaller isosurface in comparison with the H3 case, it is noticeable that an attractive interaction (blue region) is located on top of Si atoms from the V-based layer, demonstrating why the T4 configuration is the most stable.

**Figure 4:**
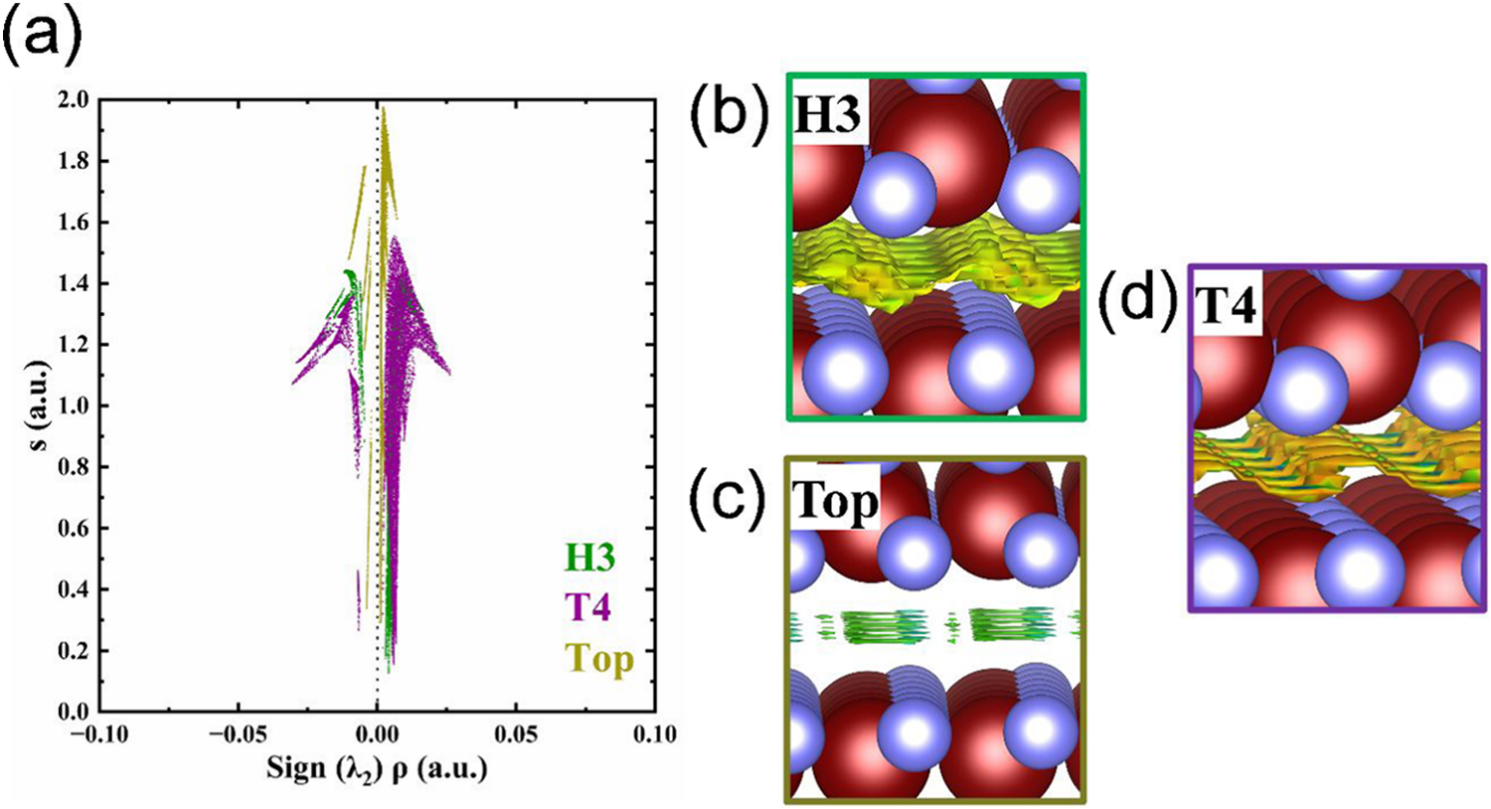
(a) *s* vs sig­(λ_2_)­ρ graph for the H3 (green), T4 (purple), and Top (yellow) stackings. The atomic models with *s* = 0.5 au isosurfaces colored in the RGB scheme are depicted in parts (b)–(d) for H3, Top, and T4 stackings, respectively.

To evidence the dynamic stability of the structure, we calculated the phonon band structure of the Sc-based layer, adopting the V-based layer cell parameter and vice versa. The results are in Figure S3a from the SI. In both cases, only positive frequencies are observed, corroborating the stability of the structure. Besides, we performed AIMD calculations at 500 K to investigate the thermal stability of the T4 structure (Figure S3b). Our results after simulation show no broken bonds or distortions, demonstrating the thermal stability of the system.

### 3.3 Magnetic Properties

Once the vdW heterostructure is well-described, we focus on its magnetic characteristics. Isolated monolayers exhibit FM characteristics, as shown in the previous section. To evaluate the magnetic coupling between V- and Sc-based materials, we considered two cases: in the first one, the spins of both layers are aligned in the same direction (FM configuration); in contrast, in the second case, the spins of both layers are aligned antiparallel (AFM configuration). The atomic representation of both configurations is shown in Figure 5.

**Figure 5:**
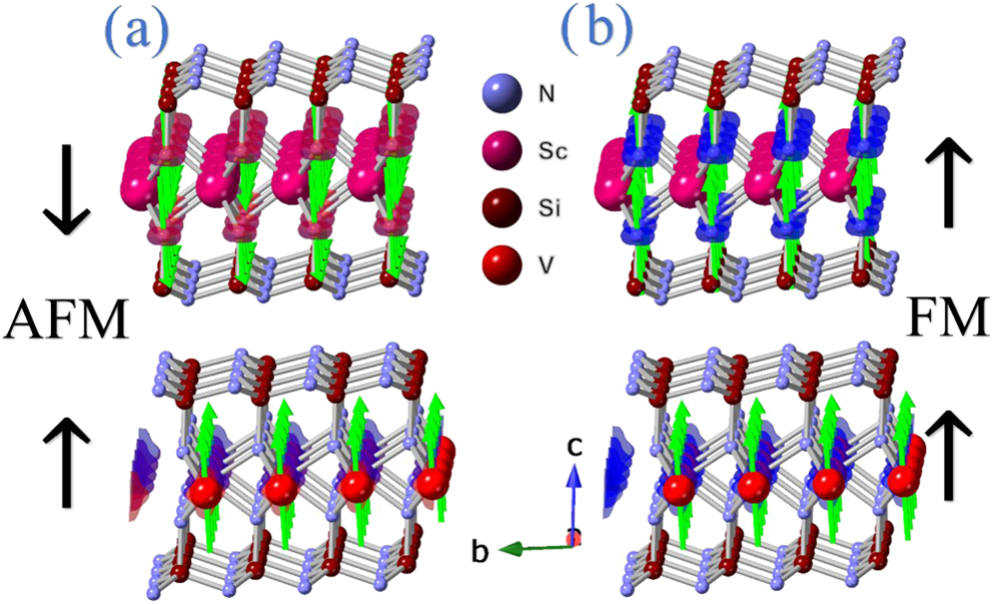
Magnetic configurations considered in this work: (a) is for AFM and (b) is for FM couplings, respectively.

The results are summarized in Table 1. The relative energies between FM and AFM configurations prove that FM coupling is more stable for the three stackings. However, the energy difference is of the order of ∼1 meV; therefore, both configurations can appear in the experiment. Notice that Top stacking has an energy difference of 0.18 meV, while H3 and T4 exhibit energy differences of 0.68 and 1.88 meV, respectively. This trend is related to the stacking between VSi_2_N_4_ and ScSi_2_N_4_ layers since the Top configuration minimizes the interaction between layers. On the other hand, T4 stacking favors the interaction between monolayers, generating a more significant energy difference between magnetic couplings.

**Table 1:** Relative Energy between FM and AFM Configurations (in meV)^a^

	relative energy (meV)		magnetic moment (μ_B_)				total magnetization (μ_B_/cell)	
stacking	FM	AFM	V	N_Sc_	V	N_Sc_	FM	AFM
T4	0.00	1.88	1.05	0.35	1.05	–0.35	6.75	0.02
				0.42		–0.42		
H3	0.00	0.68	1.05	0.36	1.05	–0.36	6.78	0.02
				0.42		–0.42		
Top	0.00	0.18	1.06	0.37	1.06	–0.37	6.84	0.03
				0.41		–0.41		

a Magnetic moments per atom and total magnetization of the T4, H3, and Top stackings.

The V atoms exhibit magnetic moments of 1.05 μ_B_ for the three different stacking and two magnetic alignments, a value similar to those observed in the isolated layer (1.07 μ_B_). The N atoms bound to Sc (N_Sc_) that form the upper-N layer (see Figure 2) have magnetic moments of ±0.42 μ_B_, depending on the FM or AFM alignment, respectively, values closer to those observed in the isolated monolayer (0.41 μ_B_). However, the N of the below-N layer reduces its magnetic moments due to the interaction with the V-based layer, regardless of the magnetic alignment. For the T4, H3, and Top stacking, we observe magnetic moments of ±0.35, ±0.36, and ±0.37 μ_B_. Note that between more interaction experiments in the layers, the reduction of the magnetic moment increases.

Magnetic anisotropy energy (MAE) is investigated for T4 stacking. To achieve this, total energies for the in-plane (100) and out-of-plane (001) magnetizations were computed. MAE is calculated as follows

1 $$E_{\mathrm{MAE}}=E_{\left(100\right)}-E_{\left(001\right)}$$

The first and second terms are the total energies of the heterostructure with the magnetization aligned in-plane and out-of-plane, respectively. Positive and negative values suggest out-of-plane and in-plane preferential magnetization, respectively. Previous reports have reported in-plane magnetization for both ScSi_2_N_4_ and VSi_2_N_4_ layers.^16^ The value obtained for the T4 heterostructure was −29.21 meV, which clarifies that easy in-plane polarization of single layers prevails.

Note that the magnetic properties and MAE are unchanged regardless of the magnetic coupling between the layers that form the heterostructure. This behavior is evidence of weak interaction between layers in the heterostructure.

### 3.4 Electronic Properties

In contrast to the structural and magnetic properties, the electronic properties of the heterostructure experience significant changes as a function of the cell parameter. Figures S4 and S5 from SI show the VSi_2_N_4_ and ScSi_2_N_4_ isolated monolayer band structures under 3 and −3% strain, respectively. About the V-based monolayer under tensile strain, the semiconductor nature is preserved. However, the band gap for spin-up is reduced from 0.54 to 0.32 eV, while the gap for spin-down remains unchanged. On the other hand, the Sc-based monolayer under compressive strain is still half-metal, with spin-down working as a metal and spin-up exhibiting a gap of 2.06 eV, ∼0.3 eV lower than the nonstressed layer.

We report the band structure and DOS of the heterostructure by considering the cell parameters of both compounds. However, since the sampling of the primitive cell is more accurate, we employed the effective band structure method^56,57^ to unfold the band structure of the supercell into the Brillouin zone of the primitive cell. In the first case, the Sc layer is under −3% strain to adopt the V layer cell parameter (−3% strain heterostructure). The second case occurs when the V layer is under tensile strain to couple to the Sc layer (3% strain heterostructure). Only the electronic properties of the most stable stackings are also investigated. Besides, we considered the FM and AFM configurations. Figure 6 shows the band structure along the Γ–M–K−Γ path and its corresponding DOS for T4 stacking in the FM configuration. In all cases, the energy reference is set at the Fermi level. Figure 6a corresponds to the −3% strain T4 heterostructure. The upper panel, from left to right, is for the band structure of the Sc-based layer, the complete heterostructure, and the V-based layer, respectively. The results evidence the metallic nature of the heterostructure. By decomposing the band structure into the contribution of each layer, we can note that the ScSi_2_N_4_ compound becomes metallic due to the majority spin channel (spin-up) shifting to upper energies at the γ point. Regarding the VSi_2_N_4_ layer, a transition from semiconductor to half-metal behavior is noticed with spin-up channels (majority spin) across the Fermi level at the γ point. In contrast, spin-down behaves as a semiconductor with an energy gap of ∼2.05 eV. The lower panel from Figure 6a displays the projected DOS for the ScSi_2_N_4_ and VSi_2_N_4_. Positive and negative values along the DOS axis are for spin-up and down, respectively. According to the results, the nitrogen atoms make the most important contribution to the DOS at the Fermi level for ScSi_2_N_4_ ML, followed by the contribution of the Sc orbitals. On the other hand, the conduction channel for spin-up in the VSi_2_N_4_ ML is mainly formed by the contribution of the V orbitals. Besides, the maximum of the valence band and the minimum of the conduction band are formed mainly by the contribution of the N and V orbitals, respectively. Figure 6b shows the band structure and the DOS for the 3% strain T4 heterostructure. Note that the heterostructure remains metallic. The Sc-based layer remains half-metallic, as in the isolated case. However, although the VSi_2_N_4_ layer becomes half-metallic, as in the previous case, a Dirac cone appears at the K point. The isolated compressive V-layer is a semiconductor with a smaller energy gap than the nonstressed layer. Therefore, we can conclude that the half-metal nature and the formation of the Dirac cone are consequences of proximity effects between layers. Also, note that the spin-up channel only has hole pockets. This break in the electron–hole symmetry has been previously observed in systems such as carrier-doped graphene interacting with Bi_2_Se_3_ ^58^ or Bi_2_Te_2_Se,^59^ N-doped graphene,^60^ and heterostructures like graphene/Si^61^ or graphene/h-BN^62,63^ and is associated with proximity effects. Our system identifies a T4-type stacking configuration previously recognized as energetically favorable in MoS_2_-based heterostructures. This stacking introduces additional band structure asymmetry, further breaking the particle-hole symmetry and contributing to the observed Dirac point shift.

**Figure 6:**
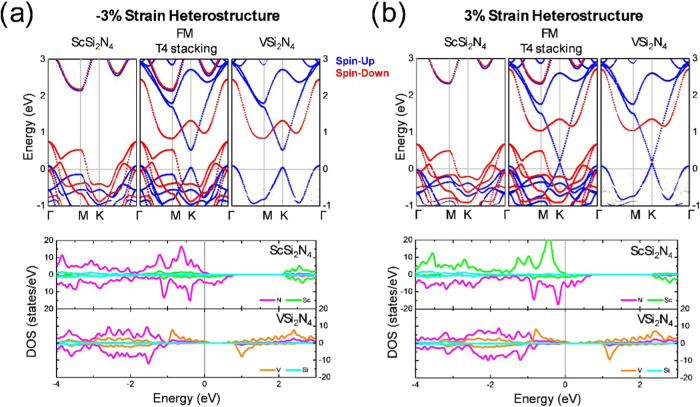
(a) DFT unfolded band structure along the Γ–M–K−Γ pathway and corresponding DOS for the −3% strain heterostructure in the T4 stacking with the FM configuration. (b) The DFT unfolded band structure along the Γ–M–K−Γ pathway and corresponding DOS for the 3% strain heterostructure in the T4 stacking with the FM configuration.

Figure 7a,b depicts the band structure and corresponding DOS for the −3 and 3% T4 heterostructures in AFM configuration, respectively. A metallic behavior is observed in the −3% strain case, similar to the FM configuration. Again, the ScSi_2_N_4_ ML becomes metallic by shifting the electronic states of spin-down to higher energies at the γ point (see the upper panel of Figure 7a). On the other hand, VSi_2_N_4_ is half-metal, as in the FM case. The projected DOS (bottom panel of Figure 7a) shows the same trend as the FM case; the main contribution to the DOS at the Fermi level for the Sc-based layer is mainly from the N orbitals. In contrast, for V-based ML, the conduction channels at the Fermi level (for spin-up) are formed by the V orbitals. Interestingly, the system acquires half-metal characteristics for the 3% strain heterostructure. By decomposing the contribution to the band structure, it is noticed that both layers are half-metallic with conduction channels for spin-up across the Fermi level. Besides, a Dirac cone that comes from V-layers is observed as in the FM case.

**Figure 7:**
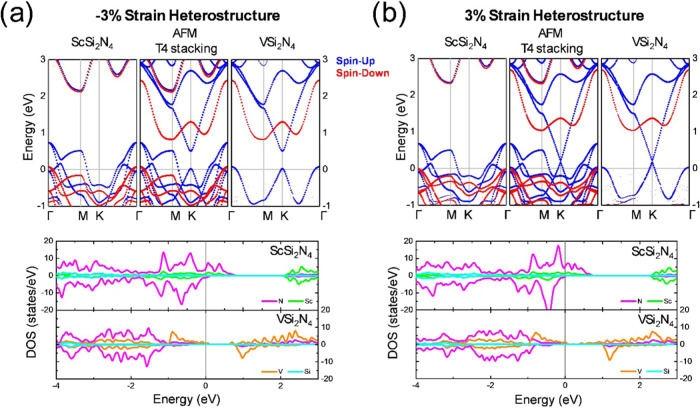
(a) DFT unfolded band structure along the Γ–M–K−Γ pathway and corresponding DOS for the −3% strain heterostructure in the T4 stacking with the AFM configuration. (b) The DFT unfolded band structure along the Γ–M–K−Γ pathway and corresponding DOS for the 3% strain heterostructure in the T4 stacking with the AFM configuration.

Our findings demonstrate that the ScSi_2_N_4_/VSi_2_N_4_ heterostructure could modulate its electronic properties as a function of the cell parameter. Once the structure acquires half-metal characteristics, it becomes suitable for implementation in spintronic devices, such as spin valves.

## 4 Conclusions

The vdW heterostructure, formed by ScSi_2_N_4_ and VSi_2_N_4_ MLs, was investigated by DFT calculations. Isolated monolayers show dynamic stability and ferromagnetic properties. Besides, the ScSi_2_N_4_ is half-metallic, and VSi_2_N_4_ is a semiconductor. Our findings show that the T4 stacking is the most favorable configuration. The NCI index is employed to investigate the interaction between ML, where it is noticed that only the vdW force participates in the interaction. Also, T4 stacking magnifies the interaction between layers, evidencing their stability. Magnetic coupling between layers is investigated, and the results show that an FM or AFM ordering could appear. Also, the MAE is calculated, and the results show an in-plane magnetization. Regarding the electronic properties, the heterostructure could be modulated from metallic to half-metallic as a function of the cell parameter and magnetic coupling. The ScSi_2_N_4_ layer works as a metal or half-metal, depending on whether the layer is with or without strain. On the other hand, VSi_2_N_4_ acquires half-metal characteristics that could be attributed to proximity effects. Our findings evidence the magnetic intrinsic nature of the structure, making it suitable for implementation in various novel spintronic devices, such as spin valves.

## Supplementary Material



## Notes

Average electrostatic potential perpendicular to the surface; band structure of isolated monolayers with and without SOC; phonon band structure for ScSi_2_N_4_ 3% strain and VSi_2_N_4_ −3% strain, and AIMD at 500 K for the T4 heterostructure; band structure of the ScSi_2_N_4_ 3% strain and VSi_2_N_4_ −3% strain (PDF)
