# Supplementary material for: Structural, Electronic, and Magnetic Properties of the van der Waals ScSi2N4/VSi2N4 Heterostructure: A First-Principles Study
Source: ACS Omega. 2025 May 20;10(21):22062–70. doi: 10.1021/acsomega.5c02195 (PMC12138823; doi:10.1021/acsomega.5c02195)
Supplement: Supplementary file 1 [file ao5c02195_si_001.pdf]

Supplementary Information

# Structural, electronic, and magnetic properties of the van der Waals $ScSi_2N_4/VSi_2N_4$ heterostructure: a first-principles study

*Brandon Pedroza-Rojas[1], Ariadna Sanchez-Castillo[2], Rodrigo Ponce-Pérez[3*]*

*[1] Universidad Autónoma del Estado de Hidalgo, Instituto de Ciencias Básicas e Ingeniería, Área Académica de Ciencias de la Tierra y Materiales, Carretera Pachuca-Tulancingo Km. 4.5, Mineral de la Reforma, Hidalgo, CP 42184, México.*

*[2] Universidad Autónoma del Estado de Hidalgo, Escuela Superior de Apan, Carretera Apan-Calpulalpan Km. 8, Col Chimalpa, Apan, Hidalgo, CP 43920, México.*

*[3] Centro de Nanociencias y Nanotecnología, Universidad Nacional Autónoma de México, Ensenada, B.C, CP 22800, México*

The average electrostatic potential perpendicular to the surface is depicted in Figure S1 for the $ScSi_2N_4$ and $VSi_2N_4$ monolayers. Our results show that a vacuum space of 10 Å is enough to avoid interactions between periodic layers.

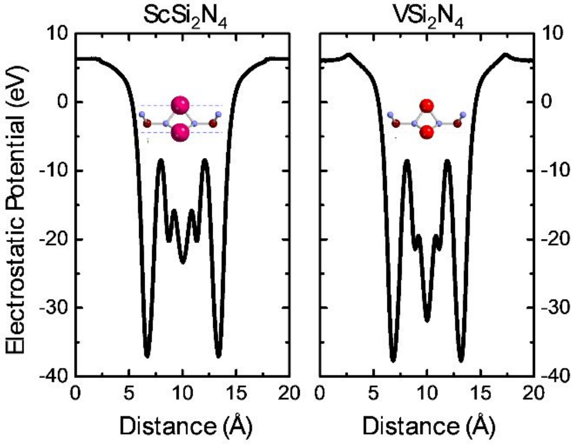


**Figure S1**. Average electrostatic potential perpendicular to the surface for the $ScSi_2N_4$ and $VSi_2N_4$ monolayers.

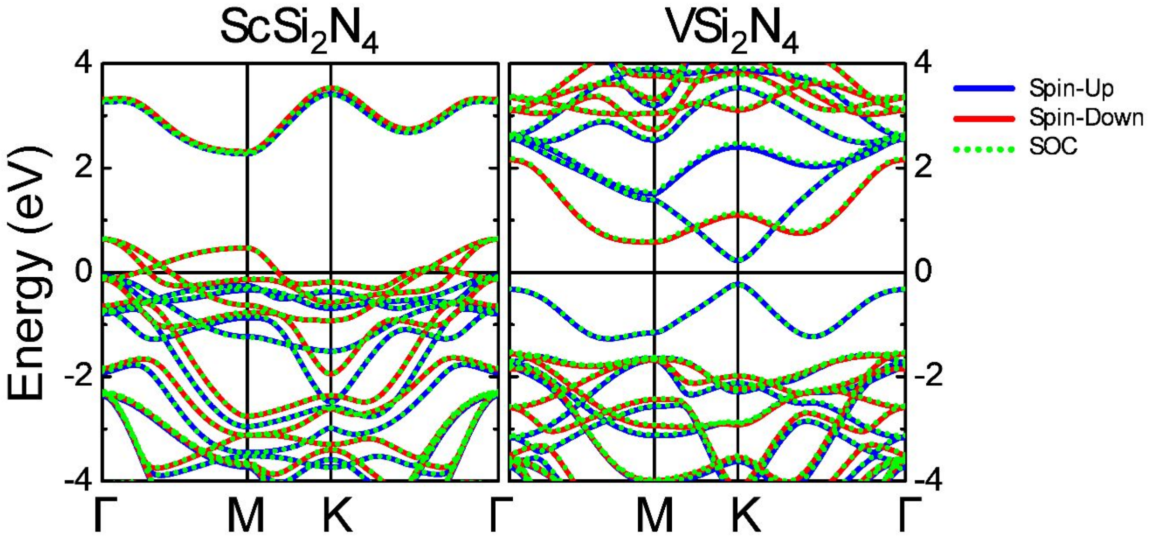


**Figure S2**. Band structure along the Γ-M-K-Γ pathway for the Sc- and V-based layers with and without SOC.

The dynamic and thermal stability of the T4 vdW heterostructure is evaluated through phonon calculations and AIMD at 500 K. The results (see **Figure S3**) show only positive frequencies denoting their stability. Besides, after the AIMD simulation, non-deformation or broken bonds are observed, which evidences their thermal stability.

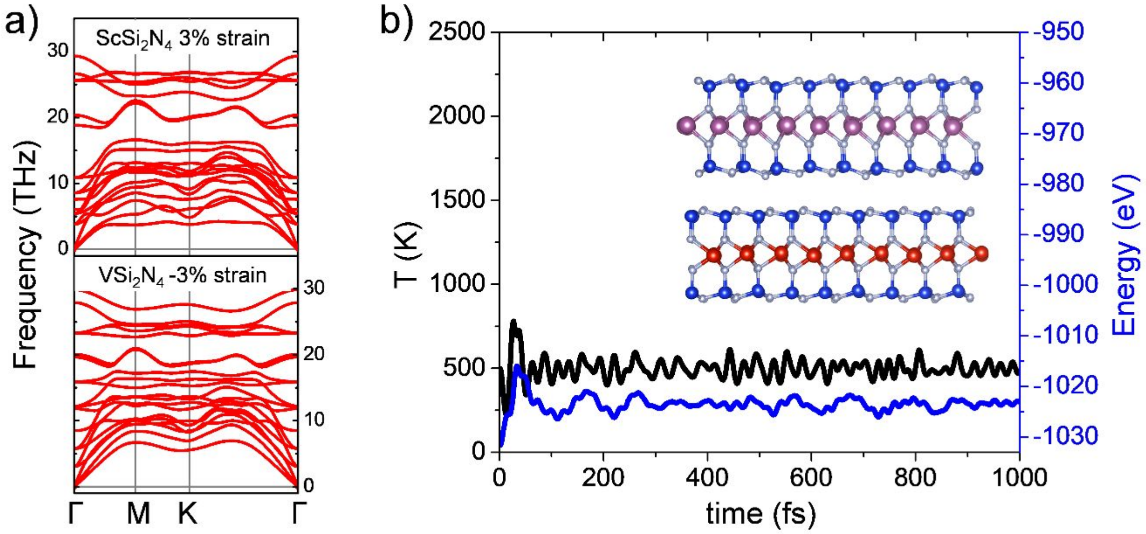


**Figure S3**. a) Phonon band structure of the $ScSi_2N_4$ and $VSi_2N_4$ under 3% and -3% of tensile/compressive strain, respectively. b) The AIMD simulation of the T4 heterostructure at 500 K is displayed; the inset shows the structure after simulation.

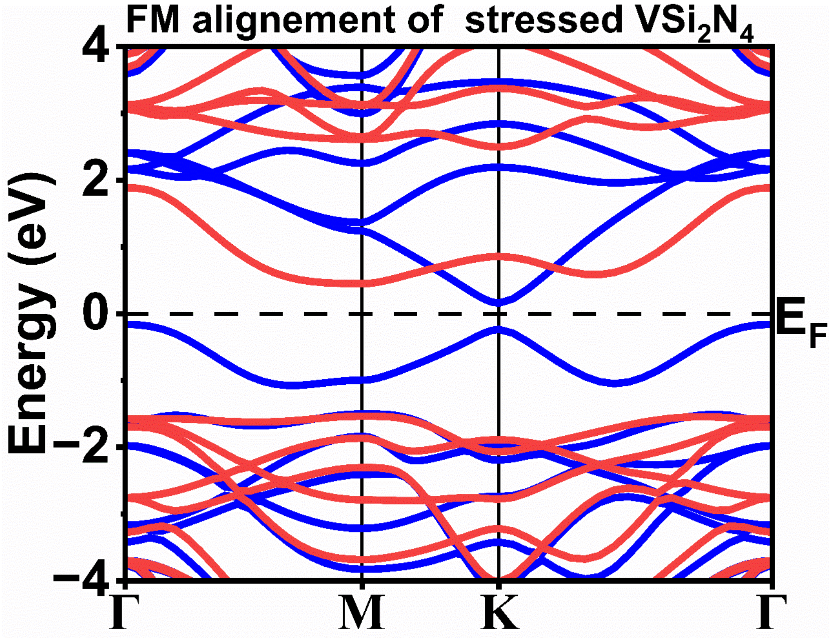


**Figure S4**. The band structure of the $VSi_2N_4$ compound under 3% of tensile strain along the Γ-M-K-Γ path. Blue lines are for spin-up and red lines are for spin-down.

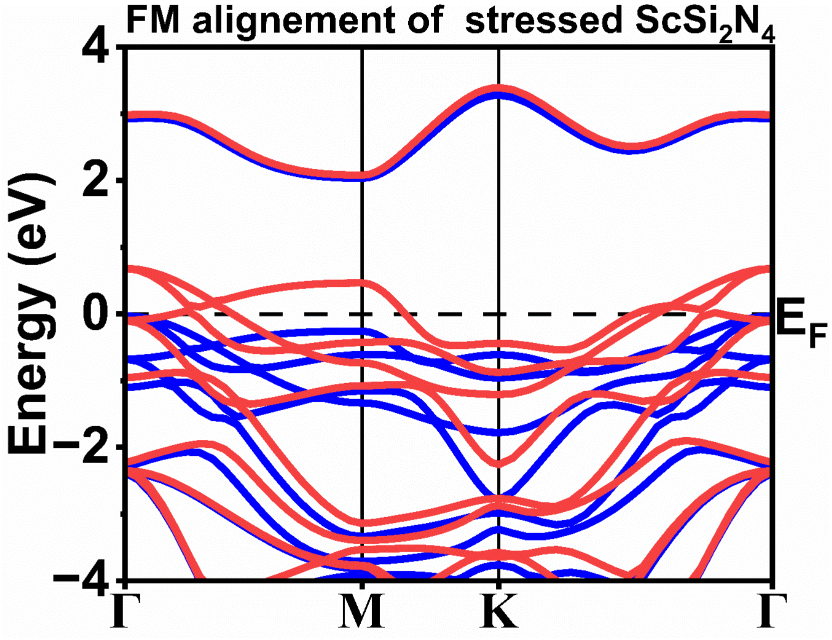


**Figure S5**. The band structure of the $ScSi_2N_4$ compound under -3% of compressive strain along the Γ-M-K-Γ path. Blue lines are for spin-up and red lines are for spin-down.
